# COVID-19 Vaccine Hesitancy in the Month Following the Start of the Vaccination Process

**DOI:** 10.3390/ijerph181910438

**Published:** 2021-10-04

**Authors:** Liviu-Adrian Cotfas, Camelia Delcea, Rareș Gherai

**Affiliations:** 1 Department of Economic Informatics and Cybernetics, Bucharest University of Economic Studies, 010552 Bucharest, Romania; liviu.cotfas@ase.ro; 2 Faculty of Medicine and Pharmacy, University of Oradea, 410073 Oradea, Romania; rares.gherai@softscape.ro

**Keywords:** COVID-19 vaccination, stance analysis, vaccine, opinion mining, vaccine hesitancy, natural language processing

## Abstract

The occurrence of the novel coronavirus has changed a series of aspects related to people’s everyday life, the negative effects being felt all around the world. In this context, the production of a vaccine in a short period of time has been of great importance. On the other hand, obtaining a vaccine in such a short time has increased vaccine hesitancy and has activated anti-vaccination speeches. In this context, the aim of the paper is to analyze the dynamics of public opinion on Twitter in the first month after the start of the vaccination process in the UK, with a focus on COVID-19 vaccine hesitancy messages. For this purpose, a dataset containing 5,030,866 tweets in English was collected from Twitter between 8 December 2020–7 January 2021. A stance analysis was conducted after comparing several classical machine learning and deep learning algorithms. The tweets associated to COVID-19 vaccination hesitancy were examined in connection with the major events in the analyzed period, while the main discussion topics were determined using hashtags, n-grams and latent Dirichlet allocation. The results of the study can help the interested parties better address the COVID-19 vaccine hesitancy concerns.

## 1. Introduction

Widespread coronavirus disease 2019 (COVID-19) has resulted in a call for action to obtain a vaccine in a short period of time [1]. This action has increased vaccine hesitancy and anti-vaccination speeches all over the world, undermining the efforts to control the spread of the novel coronavirus [2]. In a recent study, Pullan and Dey [1] have shown that throughout the pandemic interest in a coronavirus vaccine has increased and has continued to remain at a high level.

In this context, analyzing public opinion related to the hesitancy towards COVID-19 vaccination can bring new insights on the evolution of this phenomenon, as it has been observed that negative attitudes were rising on Twitter in the month preceding the start of the vaccination process in UK [3]. The increase in the number of negative and hesitant tweets was 95.28% in the days following the UK authorization of the Pfizer BioNTech vaccine (2 December 2020–8 December 2020) compared to the period 9 November 2020–1 December 2020, in which the public was aware of the existence of a vaccine. This change in the absolute number of negative and hesitant tweets was partially supported by the overall increase in the tweets posted in connection with the COVID-19 vaccination process, showing once more the attention given by the general public to this process [3].

Considering the scientific literature, vaccine hesitancy is defined as the delay in acceptance or refusal of vaccine, even though the vaccination services are available [4]. According to MacDonald [4], vaccine hesitancy comprises all the individuals with beliefs ranging between accept all vaccines with no doubts and refuse all vaccines with no doubts. Reasons for vaccine hesitancy are both complex and context-specific [2] and the uptake of a vaccine can vary depending on demographic factors such as age, gender, culture, geographic area, education, political views, socio-economic status or on factors related to the importance given to the disease, the convenience of access or the convenience of the vaccine itself [2,4,5,6].

The criteria for including an individual in a vaccination hesitancy group might be different from study to study and this issue might arise due to the ambiguity incorporated by the term “hesitancy”. As Peretti-Watel et al. [6] mentioned in a discussion paper, this term appeared as a replacement for the “vaccine resistance” or “vaccine opposition” terms, being the reason for which some of the authors have decided to include or exclude in the vaccine-hesitant group the individuals with a strong opposition to vaccination. The difference between the use of the vaccine hesitancy term has also been observed by Dube et al. [7], who underline that sometimes the eligible persons to be included in the vaccine hesitancy group comprises, besides the “unsure” individuals, even the individuals who “did not want” (“refuse”) to get the vaccine. This extended definition, which includes the persons who refuse to vaccinate despite the availability of the vaccines, has been used by various researchers [1,8,9] and even by the World Health Organization (WHO) in its 2019 report on the 10 threats to the global health [10]. In this report, the WHO underlines the importance of vaccination by pointing out that it is one of the most cost-effective measure in avoiding diseases [10]. Regarding vaccine refusal, Jacobson et al. [11] state that it is only a part the larger problem related to vaccine delay and hesitancy.

As a result, in the present paper, we will use the term vaccine hesitancy in its extended meaning by including in the hesitant persons group even the persons who refuse the COVID-19 vaccine.

Based on the scientific literature, the COVID-19 vaccination theme has captured the attention of researchers around the world both before and after the vaccination process started in UK on 8 December 2020. A series of papers have been written in this area, most of them focusing on the individuals’ opinion related to the acceptance or refusal of a vaccine.

The negative attitudes towards vaccines and the refusal to receive vaccines are pointed out as the major barriers in managing the COVID-19 pandemic in the long-run by Paul et al. [12]. According to the authors, of the 32,361 UK adults taking part of the survey related to COVID-19 vaccination, 14% mentioned unwillingness to receive a vaccine, while 22.5% marked as unsure. Another study focusing on the vaccine acceptance or hesitancy in UK conducted by Robertson et al. [2] has determined an overall vaccine hesitancy percentage of 18%, higher in the case of women, young persons, low education levels and some ethnical groups.

In the European Union, studies have been focused on the individuals’ opinion related to vaccination based on their home-residency. The reported rates for the vaccine acceptance in different studies have been: 35.3% in Portugal [13], 75% in Finland, 94.1% in Italy [14], 75% in France [15] and 59% in Slovenia [16].

A series of studies featuring US respondents have determined different rates of COVID-19 vaccine acceptance: 50% [5], 59.1% [17] 67% [18], 69% [19], 96.7% [20], being highly associated, in most of the cases, with individual socio-demographic and behavioral factors.

In Australia, the expected uptake of the vaccine has been estimated to be 86% by Borriello et al. [21], while Dodd et al. [22] reported an acceptance percentage of 76.5%.

Chinese adults’ willingness to vaccinate has been analyzed by Liu et al. [23]. The authors have shown that the introduction of the free vaccination policy has increased the willingness from 73.62% to 82.25%. The primary reasons for vaccine hesitancy have been the safety and the side effects of the novel vaccines [23].

In Israel, the country leading the vaccination race, as of 4 January 2021 (https://www.bbc.com/news/world-55514243, accessed on 20 September 2021), the opinion to take a COVID-19 vaccine has been analyzed by Shacham et al. [24]. The authors determined that dental hygienists have had a significantly higher anti-vaccination attitude than dentists and the general public. Also, it has been observed that the attitude towards COVID-19 vaccination has been more negative than towards more general vaccines [24].

A Twitter sentiment analysis was performed by Praveen et al. [25] on Indian tweets extracted for the September 2020–December 2020 period. The authors stated that 16.65% of the tweets had a negative tone regarding COVID-19 vaccination, while 35% of the tweets were positive.

Vaccination opinions dynamics from tweets in the month following the first vaccine announcement (9 November 2020–8 December 2020) have been analyzed by Cotfas et al. [3]. Overall, in the analyzed period, 17% of the tweets were against vaccination on the cumulative cleaned set comprising 752,951 tweets [3].

As presented above, most of the studies regarding the individuals’ hesitancy or acceptance for COVID-19 vaccination have been undertaken through the use of questionnaires, which have their limitations, especially related to the number of individuals considered in the study. With the rise of the social media platforms and with the increase of their use during the COVID-19 pandemic [3], in the present study the analysis will be conducted on data extracted from Twitter.

The aim of the paper is to analyze the dynamics of the public opinion on Twitter in the first month after the start of the vaccination process in UK, with a focus on the COVID-19 vaccine hesitancy messages. For this purpose, a dataset containing 5,030,866 tweets in English was collected from Twitter between 8 December 2020–7 January 2021. In this set, both original tweets and retweets from the period were included. A stance analysis was conducted using the deep learning language model RoBERTa [26], fine-tuned on a dataset containing 4341 annotated COVID-19 vaccination tweets [3]. Based on the analysis, the tweets were divided into three main categories: *in favor*, *neutral* and *against* COVID-19 vaccination. The *in favor* tweets refer to the messages which support the vaccination process, the *neutral* are mainly comprising news related to the vaccinations process, while the *against* tweets refer to vaccine hesitancy. The evolution of the three categories is analyzed in the considered period. Also, based on the unigrams, bigrams and trigrams extracted from the entire dataset, the dynamics of the *against* tweets were connected with the major event from the analyzed month.

As we are mainly interested in the dynamics of public opinion in the first month after the start of the vaccination process regarding the vaccine hesitancy, the *against* tweets are analyzed in depth by extracting the top three most used hashtags in the *cleaned* and in the *all* dataset and top six retweets. This action has the purpose of identifying the main discussion topics, which might underline the reasons behind vaccine hesitancy. Additionally, the analysis of unigrams, bigrams, trigrams and latent Dirichlet allocation on the *against* tweets dataset is used for fine-tuning the main emerging discussion topics related to vaccine hesitancy.

The paper is organized as follows: Section 2 is dedicated to the methodology and describes the data collection and analysis processes. Section 3 discusses the dynamics of public opinion related to the vaccination process, while Section 4 focuses on the vaccine hesitancy tweets. The paper closes with discussions and limitations in Section 5, conclusion and further developments are mentioned in Section 6.

## 2. Methodology

**Figure 1:**
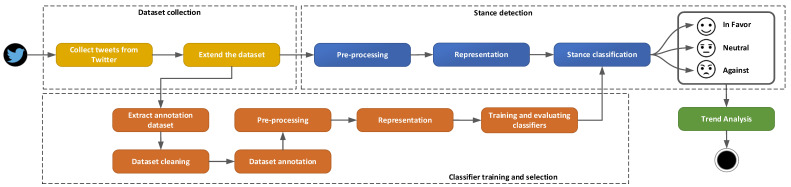
Stance detection steps.

For determining the public opinion related to the COVID-19 vaccination in the month following the start of the vaccination process in UK, the steps presented in Figure 1 have been considered.

Each of the steps needed for the stance detection are described in the following.

### 2.1. Gathering the Dataset

During this step, a language specific dataset, including only tweets written in English, is collected through the Twitter API using the keywords listed in Table 1 [3]. The dataset is then supplemented with the tweets extracted using the same keywords from the dataset provided by Banda et al. [27].

### 2.2. Classifiers Training and Selection

As suggested by D’Andrea et al. [28] and Aloufi and Saddik [29] the retweets and the duplicated tweets are removed from the dataset before the annotation process for increasing the quality of the annotated set. Given the expected high number of tweets posted in the analyzed period related to the COVID-19 vaccination, approximatively 0.3% of the tweets in the dataset are then randomly selected and manually annotated. The annotation process provides the stance for the selected tweets, which can be either *in favor*, *neutral* or *against*.

The set of tweets marked as *in favor* contains all the tweets which express positive appreciation regarding the COVID-19 vaccination process, while the set marked as *neutral* mainly features news related to the vaccination process, announcements related to the efficiency of the vaccines and the number and/or percentage of the vaccinated persons at a particular moment of time. The *against* set includes all the tweets related to the refusal to take the vaccine and the tweets in which the persons mention the fact that they will wait and learn.

In order to ensure that each annotated tweet is placed in the correct category, three persons will annotate the dataset. In case of disagreement, the class chosen by most of the annotators will be selected. As confusion between the *in favor* and the *against* category has not been previously encountered [3], it is expected that the only disagreements which might occur will be between the *in favor* and *neutral* tweets or between the *neutral* and *against* tweets.

From the annotated set, a balanced set is extracted and it is merged with the manually annotated dataset provided by Cotfas et al. [3] comprising 3249 tweets. The resulting balanced set will be used for the training and the evaluation of the classifiers.

A pre-processing step is performed on the annotated dataset through which all the user mentions, all the links and email addresses are normalized, and the emoticons are replaced with the corresponding words. Also, minor spelling mistakes are corrected, the hashtags are unpacked, the elongated words are corrected, and the letters are used in their lowercase representations. In order to perform the pre-processing step, a specific library, e.g., ekphrasis library can be used, along with the Natural Language Toolkit (NLTK) library and the “re” python module [3,30,31]. As D’Andrea et al. [28] mentioned, the pre-processing step is crucial for the success of the entire process.

Knowing that commonly very frequent words carry little “informational content”, a complex feature representation can be used for reducing the weight associated to the most frequent words. In this case, the term frequency-inverse document frequency (TF-IDF) is used with the purpose of increasing the performance of the classification algorithms that rely on the words’ frequency [3].

The classification algorithms are then trained and evaluated with the goal of determining the best classification algorithm that will be used for stance classification. The following classification algorithms are considered: Multinomial Naive Bayes (MNB) [32,33], Random Forest (RF) [34,35], Support Vector Machine (SVM) [36,37], Bidirectional Encoder Representations from Transformers (BERT) [38] and Robustly Optimized BERT Pretraining Approach (RoBERTa) [26].

The performance of the classification algorithms will be evaluated based on four widely used indicators: accuracy, precision, recall and F-score.

Accuracy indicates the ratio of correctly predicted observations to the total observations, and it is determined based on the following formula:

(1) $$Accuracy=\frac{TP+TN}{TP+TN+FP+FN}$$

where: *TP* is the number of real positive tweets classified as positive; *FP* is the number of real negative tweets classified incorrectly classified as positives; *TN* represents the number of negative tweets correctly classified as negative and *FN* is the number of real positive tweets incorrectly classified as negative.

Precision represents the ratio of correctly predicted positive observations to the total predicted positive observations:

(2) $$Precision=\frac{TP}{TP+FP}$$

Recall is the ratio of correctly predicted positive observations to all the observations in the actual class:

(3) $$Recall=\frac{TP}{TP+FN}$$

F-*Score* is computed as a weighted average as presented below:

(4) $$F-score=2\cdot \frac{Precision\;\cdot \;Recall}{Precision+Recall}$$

### 2.3. Stance Detection

The remainder of the COVID-19 vaccination dataset—obtained after the tweets to be annotated have been extracted—was subjected to a similar pre-processing and representation step as for the annotated tweets.

The resulting set was then analyzed using the best performing algorithm selected based on the values recorded for accuracy, precision, recall and F-score. The tweets belonging to each of the *in favor*, *neutral* and *against* category were then analyzed, with a focus on the tweets listed under the *against* category as these tweets reflect the general public hesitancy towards the COVID-19 vaccine, as presented in Section 3.

Additionally, in Section 4, an n-gram analysis was performed on the *against* category tweets by extracting the unigrams, bigrams and trigrams which could offer more insight on the discussion and reasons stated on Twitter by the individuals in the COVID-19 vaccine hesitancy category. The discussion topics extracted from the hashtags in the *against* category were analyzed for better observing the main reasons behind the hesitancy speech. Also, the vaccine hesitancy topics were uncovered with the help of a latent Dirichlet allocation analysis (as presented in Section 4).

## 3. COVID-19 Vaccine Stance Dataset and Stance Detection

For the period 8 December 2020–7 January 2021 a number of 5,030,866 tweets in English have been extracted. After all the retweets and duplicated tweets were discarded, a *cleaned* dataset containing 1,221,694 tweets were obtained.

The evolution of the cleaned tweets and of the tweets in the mentioned period is depicted in Table 2.

**Figure 2:**
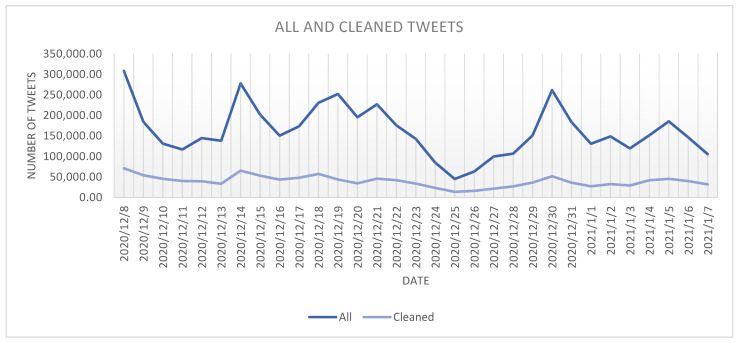
The evolution of *all* and *cleaned* tweets in the analyzed period.

As it can be observed from Figure 2, there have been a series of “spikes” in the number of tweets published in the analyzed period. The presence of the spikes is better observed in the case of the *all* dataset, as in the days characterized by the existence of such spikes the number of tweets in the *all* dataset is higher with up to 577.55% than the number of tweets in the cleaned dataset.

The correspondence between the spikes and the events that took place in the days characterized by these spikes will be further analyzed in Section 4 of this paper.

### 3.1. COVID-19 Vaccine Stance Dataset

From the *cleaned* dataset, a sample containing 3657 tweets has been randomly extracted, representing 0.3% of the *cleaned* dataset. These tweets have been manually annotated by three evaluators, in an independent manner. When confronting the results of the annotation process from the three evaluators, disagreements have been encountered only in few cases among the *in favor* and *neutral* tweets or among the *neutral* and *against* tweets. No situations have been encountered when a tweet has been annotated as both *in favor* and *against.* When disagreements have been observed, the class chosen for the tweets has been the one selected by most of the annotators.

The statistics regarding the distribution of the *in favor*, *neutral* and *against* tweets in the annotated dataset are presented in Table 3.

This set has been extended by considering the dataset of 7530 annotated tweets provided in [3] (1083 against, 5188 neutral and 1259 in favor), collected for vaccine hesitancy between 9 November and 8 December 2020, which has conducted to an annotated dataset of 11,187 tweets (1447 against, 7830 neutral and 1910 in favor), representing 0.9% of the *cleaned* dataset extracted for this paper.

As a result, the extended balanced dataset selected from this combined dataset comprises 4341 tweets, that have been used for evaluating the performance of the classification algorithms.

### 3.2. COVID-19 Vaccine Stance Detection

Five classification algorithms have been considered: MNB, RF, SVM, BERT and RoBERTa. A grid search approach has been used for determining the best parameters for the developed natural language processing pipeline.

In the case of the classical machine learning algorithms (C1-C6), namely MNB, RF and SVM different n-gram combinations have been investigated for the string vectorizer, ranging from (1, 1), corresponding to unigrams, to (1, 3), corresponding to unigrams, bigrams and trigrams. The algorithms have been evaluated by considering two situations: the one in which general stop words are removed using the stop words list provided by the Natural Language Toolkit NLTK library [31] and the one in which corpus-specific stop words are removed using as document frequency thresholds 0.5, 0.75 and 1.0. The possibility of using the TF-IDF for improving the stance classification accuracy has been analyzed, as well as limiting the total number of features to 1500, 2000 and 3000. Different settings for the classifiers’ parameters have been considered, including varying the alpha parameter and the penalty in the case of the SGDClassifier, corresponding to a linear SVM.

The results achieved using the parameters determined through grid search (C1, C3 and C5) and those corresponding to the n-gram model (1, 3), which includes unigrams, bigrams and trigrams, while keeping all the features, have been included in Table 4. It can be observed that for all three algorithms the best results have been achieved when including unigrams and bigrams, without excluding the general stop words. The accuracy of best performing classifier in each category has been highlighted in bold in Table 4.

In the case of the Multinomial Naïve Bayes classifier (C1 and C2) the best results have been achieved for the C1 classifier, for which the maximum number of features has been reduced to 3000, without excluding corpus specific stop words, corresponding to a document frequency threshold of 1.0.

The Random Forest classifier (C3 and C4) has reached the best performance when all the features have been included, but the context specific stop words have been excluded using a document frequency threshold of 0.5.

For the Support Vector Machines classifier (C5 and C6) the most accurate results have been obtained without limiting the number of features or excluding stop words, while setting the alpha parameter at 0.00001 and choosing an “elasticnet” regularization penalty.

The best performing classical machine learning classifier has been C5, a SVM classifier, having an accuracy of 72.19%. It has achieved better F-scores than the other classifier on all three classes, *against*, *neutral* and *in favor*.

In the case of the deep learning algorithms (C7–C9), namely BERT and RoBERTa, the best values for the learning rate, batch size and number of epochs hyper parameters have been determined following the approaches recommended by Devlin et al. [38] and Liu et al. [26]. Thus, for both models the considered batch sizes have been 16 and 32, while the number of epochs has been 2, 3 and 4. The analyzed learning rates have been $2\times 10^{-5}$ , $3\times 10^{-5}$ and $5\times 10^{-5}$ for BERT [38] and $10^{-5}$, $2\times 10^{-5}$ and $3\times 10^{-5}$ for RoBERTa [26]. Both the cased and the uncased versions of the BERT language model have been considered. The cased version differs from the uncased one, by considering the casing of the letters.

In the case of the BERT algorithm (C7 and C8), the best results have been reached when the learning rate has been set to $5\times 10^{-5}$, the batch size to 32 and number of epochs to 4. The uncased version of the BERT algorithm (C7) has provided a better accuracy than the cased version (C8).

The best performing deep learning classifier has been RoBERTa (C9), which provides both better accuracy and a better F-Score when compared to C7 and C8 algorithms. The best results for RoBERTa have been achieved by choosing a learning rate equal to $2\times 10^{-5}$, a batch size of 16 and a number of epochs equal to 4.

Overall, based on the results provided in Table 4 it can be observed that the best performing classifier has been RoBERTa (C9) having an accuracy of 78.63%. The F-score for the three considered classes, also exceed those for all the other algorithms. As a result, C9 has been used in the following for the stance analysis on both *all* and *cleaned* datasets.

#### 3.2.1. Cleaned Tweets Stance Analysis

**Figure 3:**
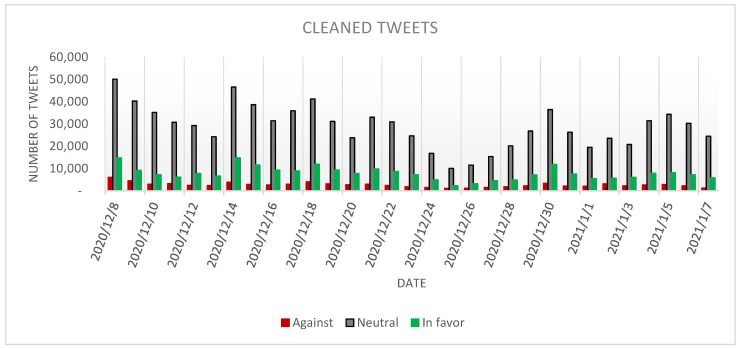
The evolution of the *cleaned* tweets stance.

The *cleaned* tweets stance analysis has been conducted using C9 classifier and the results are presented in Figure 3.

From Figure 3 it can be observed that most of the tweets belong to the *neutral* category (894,664 tweets representing 73.23% of the dataset), an expected outcome given the high number of *neutral* tweets in the manually annotated dataset (Table 3), while the second-large category is represented by the *in favor* tweets (244,159 tweets representing 19.99% of the dataset). The *against* tweets are the least represented in the dataset (82,871 tweets amounting to 6.78% of the dataset).

It can be observed that the day with the most tweets in all the categories (50,087 *neutral*, 14,839 *in favor* and 6084 *against*) has been 8 December 2020.

Even in this case, it can be noticed that there are few spikes across the analyzed period, which can be encountered at a larger scale in the case of the *neutral* tweets, followed by spikes of a smaller magnitude in the case of the *in favor* and *against* tweets.

When comparing the results of the present study with the one performed by Cotfas et al. [3], which analyses the tweets published in the month prior to the start of the vaccination process, it can be observed that the proportion of the *neutral* tweets has increased from 66% in [3] to 73.23% in the current study. Regarding the *in favor* tweets it can be noticed that there was a slight increase from 17% to 19.99%, while the percentage of *against* tweets decreased from 17% to 6.78%.

#### 3.2.2. Entire Tweets Stance Analysis

**Figure 4:**
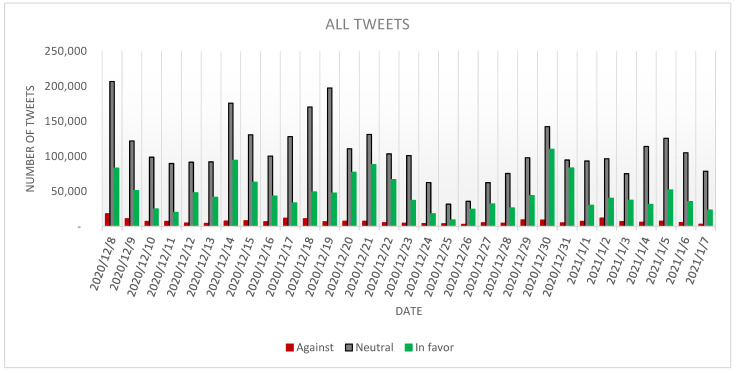
The evolution of the *all* tweets stance.

In the case of *all* tweets dataset, it can be observed (Figure 4) that the *neutral* tweets continue to be the largest category with a total of 3,342,543 tweets (66.44%), followed by *in favor* tweets, counting 1,466,971 tweets (29.16%) and by *against* tweets, counting 221,352 tweets (4.40%).

Comparing the percentages of each category in *all* and *cleaned* datasets, it can be mentioned that one can observe a decrease in the proportion held by the *neutral* and *against* tweets, and an increase in the percentage of the *in favor* tweets. As in the previous case, it can be noticed that there are a series of spikes even in the *all* dataset. The highest number of *neutral* and *against* tweets were posted on 8 December 2020 (206,640 tweets, respectively 18,212 tweets), while the day with the most *in favor* tweets was 30 December 2020, followed by 31 December 2020 (110,092 tweets, respectively 83,506 tweets).

Compared to the stance analysis results in [3], it can be observed that the percentage of *neutral* tweets decreased from 70% to 66.44%, while the percentage of *in favor* tweets increased from 20% to 29.16%. Correspondingly, the percentage of *against* tweets diminished from 10% to 4.4%.

## 4. COVID-19 Vaccine Hesitancy Analysis

In order to analyze the vaccine hesitancy, the tweets marked with *against* both in the *cleaned* and in the *all* dataset have been selected and discussed in the following section.

### 4.1. Vaccine Hesitancy Tweets Spikes Analysis

**Figure 5:**
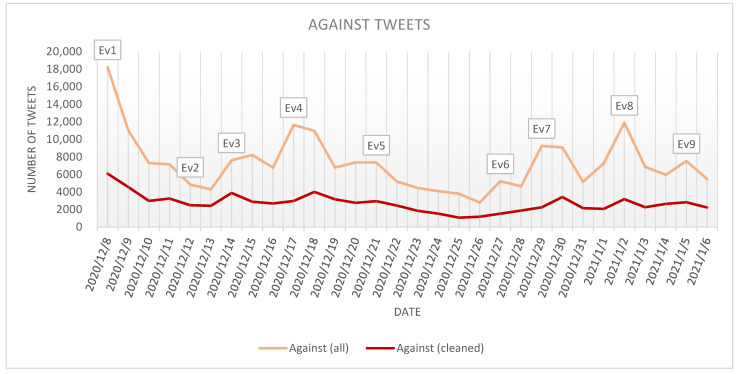
Evolution of the *against* tweets stance.

The *against* tweets in *cleaned* and *all* datasets have been plotted to better observe if there were spikes in the number of collected tweets on some particular days of the analysis. Based on Figure 5, it can be noticed that the *cleaned* dataset seems to follow the spikes in the *all* dataset, but at a smaller magnitude.

In order to identify the major events which could be put in connection to the spikes in the two datasets, the Google.com search engine was used by accessing the “News” section and typing “COVID-19 vaccination” keywords for the days in which spikes were identified. For each search, the first three pages of News were considered (where available) and the most relevant event was identified. As a note, it should be stated that in all the days of the analyzed period there was news related to COVID-19 vaccination. This situation was expected as the selected period has been marked by major events related to the start of the vaccination in different parts of the world.

A total of nine spikes were detected in Figure 5.

The associated events extracted from the News section of google.com are:

Ev1. Dec. 8: UK starts COVID-19 vaccination with Pfizer (bbc.com/news/uk-55227325 (accessed on 12 May 2021))
Ev2. Dec. 12: The Food and Drug Administration (FDA) authorizes Pfizer’s vaccine (cbsnews.com/news/fda-approves-pfizer-vaccine-emergency-use-fight-covid-19 (accessed on 12 May 2021))
Ev3. Dec. 14: US starts COVID-19 vaccination with Pfizer (abcnews.go.com/US/us-administer-1st-doses-pfizer-coronavirus-vaccine/story?id=74703018 (accessed on 12 May 2021))
Ev4. Dec. 17: FDA plans to approve the use of Moderna vaccine (apnews.com/article/coronavirus-pandemic-coronavirus-vaccine-4b34a3ffaf501bcb5300984f98cca757 (accessed on 12 May 2021))
Ev5. Dec. 21: US President-elect Joe Biden gets vaccine (bbc.com/news/world-us-canada-55401706 (accessed on 12 May 2021))
Ev6. Dec. 27: EU begins mass vaccination campaign (npr.org/sections/coronavirus-live-updates/2020/12/27/950586980/eu-begins-its-vaccine-rollout-with-goal-of-inoculating-450-million-against-covid (accessed on 12 May 2021))
Ev7. Dec. 29: Kamala Harris receives first dose of Moderna vaccine (cnn.com/2020/12/29/politics/kamala-harris-covid-vaccine/index.html (accessed on 12 May 2021))
Ev8. Jan. 2: Vaccination dry run in India (theguardian.com/world/2021/jan/02/india-prepares-for-vast-covid-vaccination-push-ahead-of-astrazeneca-oxford-jab-approval (accessed on 12 May 2021))
Ev9. Jan. 5: Vaccination progress (https://www.bbc.co.uk/news/health-55553072 (accessed on 12 May 2021))

For validating that the selected news has been representative for the mentioned days, we have extracted for each date the bigrams and trigrams from the *cleaned* dataset (in which we have kept all the tweets: *neutral*, *in favor* and *against*) and we have ordered them in accordance with the number of appearances. After excluding the specific COVID-19 bigrams and trigrams that appeared in all the days and which were not related to specific news, but to the COVID-19 vaccination process in general (e.g., “covid 19”, “19 vaccine”, “covid 19 vaccine”, “covid19 vaccine”, “coronavirus vaccines”), a series of specific bigrams and trigrams have been identified:

Ev1. Dec. 8: “year old”—5537; “90 year”—5391; “pfizer covid 19”—5326; “first person”—5202; “90 year old”—5008; “becomes first”—3797; “receive Pfizer”—3225; “vaccine uk”—2048;
Ev2. Dec. 12: “pfizer covid”—2504; “pfizer covid 19”—2437; “emergency use”—1956; “pfizer biontech”—1441; “vaccine emergency”—1382; “first covid”—1135; “first covid 19”—1123; “approves Pfizer”—1093; “19 vaccine emergency’—1036; “fda approves”—894; “fda approves Pfizer”—821;
Ev3. Dec. 14: “New York”—3501; “first covid”—3476; “first covid 19”—3371; “receive covid”—2754; “receive covid 19”—2659; “among first”—2303; “pfizer covid”—2214; “pfizer covid 19”—2144;
Ev4. Dec. 17: “moderna covid”—1739; “moderna covid 19”—1667; “emergency use”—1168;
Ev5. Dec. 21: “joe biden”—2075; “president elect”—1591; “biden receives”—1362; “first dose”—1315; “elect joe”—1183; “elect joe biden”—1181; “president elect joe”—1166;
Ev6. Dec. 27: “european union”—460; “vaccine rollout”—413; “mass covid”—327; “mass covid 19”—320; “vaccination campaign”—295; “vaccine campaign”—280;
Ev7. Dec. 29: “kamala harris”—1396; “first dose”—788; “moderna covid”—779; “moderna covid 19′—766; “harris receives”—716; “vice president”—654; “vice president elect”—604; “kamala harris receives”—585;
Ev8. Jan. 2: “dry run”—1103; “bjp vaccine”—379; “india approves”—286; “akhilesh yadav”—177; “take bjp”—51;
Ev9. Jan. 5: “vaccine rollout”—1656; “covid 19 vaccinations”—1007; “vaccine doses”—802; “vaccine distribution”—802; “get vaccine’—590; “receive covid’—508; “receive covid 19”—491; “19 vaccine rollout”—484.

Considering the *against* tweets published in the 9 days which have generated spikes in data, it can be observed that 83,538 tweets were posted, 28,161 of them being original tweets. Compared to the average number of tweets posted per day in the analyzed period (approximatively 7140 tweets per day in the *all* dataset and 2673 tweets in the *cleaned* dataset), in the 9 days for which events were identified, the increase in the number of tweets was 129.99% in the case of *all* tweets dataset and 117.05% in the case of *cleaned* tweets dataset.

The event which has generated the greatest spike in the *against* tweets is Ev1 that marks the start of the vaccination campaign with Pfizer in UK. As a result, on 8 December 2020, 18,212 *against* tweets have been posted (representing 8.23% from the total number of tweets posted in the analyzed period). Considering only the cleaned *against* tweets, a number of 6084 tweets have been identified (representing 7.34% from the total number of tweets posted in the analyzed period).

The events with the second and with the third-major impact on the increase of the number of tweets have been Ev8 characterized by the dry run on the vaccination in India (11,890 tweets in the *all* dataset) and Ev4 related to the FDA decision to approve the use of Moderna vaccine (11,641 tweets in the *all* dataset). These two events also created spikes in the *cleaned* dataset. Comparing the effect of the two events on the public opinion in the day following the occurrence of the event, it can be observed that while for Ev8 the number of tweets posted in the following day was reduced with 44.26% on the *all* dataset, in the case of Ev4 the reactions in the following day continued to appear, the number of tweets recorded in the following day being only 5.83% smaller than in the day of Ev4. The persistence of the discussions in the day after the event in the case of Ev4 was observed both in the *all* and in the *cleaned* datasets, highlighting the importance of the event for the Twitter users.

An event with a high impact on the number of *against* tweets in the *all* dataset was Ev7 related to the fact that Kamala Harris received the first dose of Moderna vaccine (9253 tweets). With all these, considering the number of the cleaned tweets recorded in the day of this event, it can be observed that the event had a lower impact on the individual tweets.

Moderate spikes have been produced by: Ev3 related to the start of the vaccination with Pfizer in US (7617 tweets in *all* dataset), Ev9 related to updates on the vaccination progress (7523 tweets in *all* dataset) and Ev5 related to the fact that the US President receives the vaccine (7352 tweets in *all* dataset). All three events (Ev3, Ev9 and Ev5) have produced spikes in the *cleaned* dataset.

Lower spikes have been produced by Ev6 which marks the start of the vaccination campaign in the EU (5220 tweets in the *all* dataset) and Ev2 regarding the authorization of the Pfizer vaccine by the FDA (4830 tweets in the *all* dataset).

### 4.2. Examples of Vaccine Hesitancy Tweets and Themes

Table 5 presents a brief selection of *against* tweets, randomly selected from the *cleaned* dataset. These tweets have been chosen for providing an evidence on the type of discourse carried by these messages. As can be observed, the authors of the tweets are expressing a series of doubts related to COVID-19 vaccination process related either to the fact that the vaccine has been produced in a short period of time, or that, as a result of the vaccination process, some changes could take place worldwide or in the body. On the other hand, there are tweets in which the authors of the tweets argue that they would better trust their immune system, while others would have been willing to take the vaccine but, as severe allergies might occur, they decide to wait for the moment.

### 4.3. Vaccine Hesitancy Hashtag Analysis

The hashtags associated with the *against* tweets have been extracted from both the *cleaned* and *all* datasets. Even in this case, some of the most used hashtags were general hashtags related to COVID-19 and vaccination, which could have been encountered also in the *in favor* and *neutral* tweets.

As we are interested in determining the most used hashtags in the analyzed period for the *against* tweets, the general hashtags that were encountered also for the *in favor* and *neutral* tweets have been eliminated and we have kept in analysis the top three hashtags from each of the *cleaned* and *all* datasets. The results are summarized in Table 6.

#### 4.3.1. Top-3 Hashtags in Cleaned Dataset Analysis

**Figure 6:**
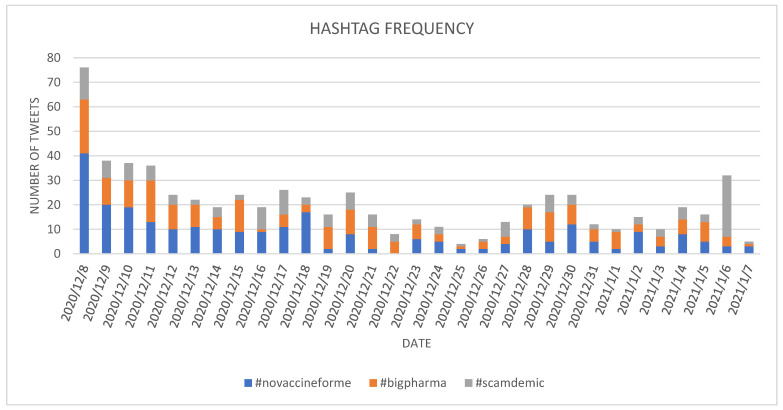
Evolution of the selected *against* hashtags in the *cleaned* dataset.

Considering the top three hashtags in the *cleaned* dataset (Figure 6) it can be observed that #novaccineforme and #bigpharma were mostly used on 8 December 2020 (counting for 63 tweets), while #scamdemic had the higher number of postings on 6 January 2021, (25 tweets), a day after Ev9 marking the update on the vaccination process worldwide. In the remaining days of the analyzed period, an average of 19 tweets per day containing one of the selected hashtags can be determined.

The use of the selected hashtags has been different in the analyzed period. While #novaccineforme has been mostly used in the first part of the period (after Ev1) and has had two spikes after the occurrence of Ev4 characterized by the FDA plan to approve the Moderna vaccine and Ev6 related to the start of the vaccination campaign in EU, #bigpharma had three spikes of a lower intensity, with one more than #novaccineforme in the days following Ev3 related to COVID-19 vaccination start with Pfizer in US.

In the case of #scandemic, besides the spike after the Ev9, two spikes of lower intensity were observed after Ev1—the start of COVID-19 vaccination with Pfizer in UK and after Ev3—the start of COVID-19 vaccination with Pfizer in US.

An analysis of the tweets containing the three selected hashtags was conducted with the purpose of extracting the most common hesitancy reasons associated with each hashtag.

Based on the issues reported by the Twitter users, the discussion topics have been divided into 9 main categories:

- Mistrust—not trusting the vaccine due to various reasons;
- Freedom—the right of each person to choose for himself/herself;
- Side effects—various side effects that have been reported or are possible outcomes of the vaccine;
- Hiding relevant information—not presenting parts or entire information related to vaccine;
- Unsafety—lack of guarantees from the authorities or pharmaceutical companies;
- Inefficiency—the protection degree of the vaccine against COVID-19;
- Existence of alternatives—different schemes of treatment or no treatment at all due to high recovery rate from COVID-19;
- Scam—reasons and facts behind the entire vaccination campaign which show that the pandemic is overrated;
- Moral and religious issues—the existence of some ingredients in the vaccine that make it impossible to be administered to certain persons due to moral or religious issues.

In the case of #novaccineforme a series of discussion topics and issues related to COVID-19 vaccination hesitancy were determined, as presented in Table 7.

Most of the reported issues were connected to side effects and mistrust in the capacity of the in-charge persons and authorities to produce a vaccine in such a short amount of time. Also, a series of issues are dedicated to proving that the entire vaccination campaign is a Scam.

Some issues were reported on hiding relevant information and the existence of alternatives, while fewer reported issues have been in the area of vaccine unsafety (Table 7). Inefficiency and moral and religious issues were also pointed out.

Table 8 presents the discussion topics and issues for #bigpharma. Three of the main topics identified above are missing from the discussion topics, namely freedom, inefficiency and moral and religious issues.

Some of the main discussion topics referred to the existence of alternatives such as pills for boosting the immune system and to the fact that the entire pandemic was a scam designed for the pharmaceutical companies to earn money. Also, the pharmaceutical companies were criticized for the fact that they are not responsible in any way for the wrongful injuries caused by the vaccine administration.

A series of side effects were discussed, both on short and long-term along with examples of situations in which different persons have died as a result of the vaccine.

In the case of #scamdemic the discussion topics with the highest variability were mistrust, side effects, existence of alternatives and scam (Table 9). Besides the discussion topics encountered in the case of #bigpharma, in the case of #scandemic a new discussion topic arose related to Moral and religious issues which might prevent the persons who want to take the vaccine to actually taking it. Two reasons were mentioned, one related to the presence of gelatin in the vaccine and another one related to the evidence of DNA from aborted fetuses. The presence of aborted fetuses was also mentioned among the issues listed in the #novaccineforme. The list of discussion topic and issues is presented in Table 9.

#### 4.3.2. Top-3 Hashtags in All Dataset Analysis

**Figure 7:**
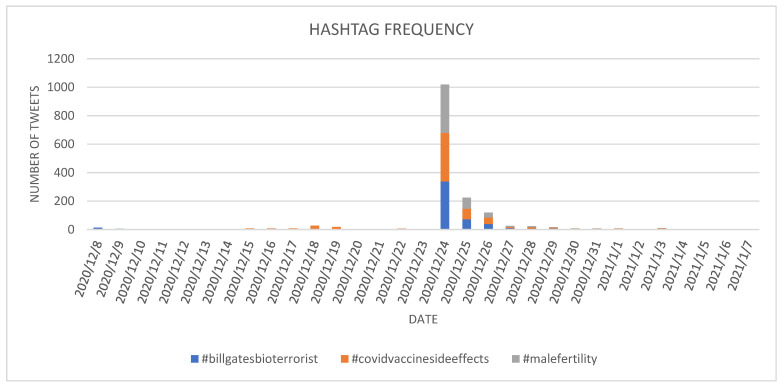
Evolution of the selected *against* hashtags in the *all* dataset.

In the case of *all* dataset, three of the most-used hashtags related to vaccine hesitancy were #billgatesbioterrorist, #covidvaccinesideeffects and #malefertility. Considering their evolution in the analyzed period it can be observed that their high usage occurred on 24 December 2020 (Figure 7)–338 tweets containing #billgatesbioterrorist, 314 tweets containing #covidvaccinesideeffects and 340 tweets containing #malefertility.

Besides this major spike, a minor spike was reported for #billgatesbioterrorist in the day of Ev1 marking the start of COVID-19 vaccination with Pfizer in UK, while for the #covidvaccinesideeffects the only other spike (besides 24 December 2020) was on the day after Ev4 related to FDA plans to approve the Moderna vaccine.

The #malefertility had only one spike on 24 December 2020, with no previous posting before the mentioned date.

After 24 December 2020, the use of the three hashtags decreased until 4 January 2021, when no tweet with the mentioned hashtags was reported.

**Figure 8:**
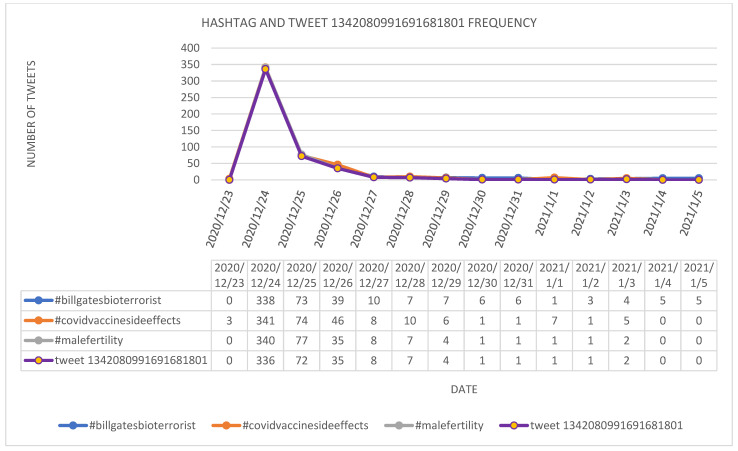
Evolution of the selected *against* hashtags and selected tweet between 23 December 2020–5 January 2021.

As the major spike in the three hashtags has not been connected to any of the events listed above, a more in-depth analysis of the tweets posted on 24 December 2020 containing the selected hashtags has been conducted. Based on the analysis it was observed that the great majority of the tweets wee, in fact, retweets of the tweet with the ID 1342080991691681801, which contained the following message: “*This IS NOT a joke! Please for the love of God & family, don’t get this vaccine. Study investigates effects of COVID-19 vaccine on #malefertility #CovidVaccinesideeffects #BillGatesBioTerrorist https://t.co/tnSPl6BQc3*”. The tweet along with the retweets were posted 336 times on 24 December 2020 and reposted by 132 times on 25 December 2020–3 January 2021. The evolution of the selected tweets and of the tweet with ID 1342080991691681801 is provided in Figure 8.

It was observed that all the retweets containing #malefertility (475 retweets) were retweets of the tweet with the ID 1342080991691681801 (467 retweets plus the original message), while the rest have been retweets of the tweet with ID 1342118751483912192 (8 retweets plus the original message) having the text: *“#MakeThisViral Covid Vaccine damages #malefertility.#TopCrims in MSM stay silent while carnage rolls out. @nytimes @NYTScience @washingtonpost @latimes @chicagotribune @AtlantaJournal @theatlantic @newscientist @MotherJones @thenation @sciam @nature @BostonGlobe @bostonherald https://t.co/FRcLxfDya3*”.

By checking the links posted in the two tweets, it has been observed that the link in the tweet with the ID 1342118751483912192 is a link to the other tweet (ID 1342080991691681801), while the link in tweet with the ID 1342080991691681801 is to a neutral tweet in which there is a link to an article posted on 20 December 2020, having the title: “*Study investigates effects of COVID-19 vaccine on male fertility*” (https://www.local10.com/news/local/2020/12/20/study-investigates-effects-of-covid-19-vaccine-on-male-fertility/ (accessed on 6 June 2021)). Even though the information in the article is positive related to COVID-19 vaccination, as the authors are citing Dr. Ramasamy who says that: “*We’re evaluating the sperm parameters and quality before the vaccine and after the vaccine. From the biology of the COVID vaccine we believe it shouldn’t affect fertility but we want to do the study to make sure that man who want to have kids in the future to assure them it’s safe to go ahead and get the vaccine*,”, the information is “deformed” when presented in the tweets with the ID 1342080991691681801, being put in a negative form. Lastly, it is worth mentioning that both accounts which posted the two tweets were suspended.

Related to the discussion topics and issues highlighted in the remainder of the tweets and retweets posted in the analyzed period under the #billgatesbioterrorist, #covidvaccinesideeffects and #malefertility, an analysis has been conducted by considering the nine discussion topics identified in the cleaned dataset (namely mistrust, freedom, side effects, hiding relevant information, unsafety, inefficiency, existence of alternatives, scam and moral and religious issues).

As mentioned above, for the #malefertility the only discussion topic was related to the male fertility (included in the side effects category).

For the #billgatesbioterrorist besides the male fertility, few other discussion topics and issues were identified in the remainder 97 tweets and retweets (besides the tweet with the ID 1342080991691681801 and its retweets) as presented in Table 10, referring to either side effects or scam.

In the case of #covidvaccinesideeffects the remainder of 92 tweets that did not contain the tweet with ID 1342080991691681801 and its retweets, approached various discussion topics and issues as presented in Table 11. As expected from the name of the hashtag, the most debated theme was related to the side effects, a series of examples of side effects being presented along with examples of specific cases in which some side effects occurred as a result of the COVID-19 vaccination. Some other discussion topics referred to: mistrust, unsafety, hiding relevant information and existence of alternatives (Table 11).

### 4.4. Vaccine Hesitancy Top Six Retweets Analysis

Considering the *all* dataset, the top six tweets that were mostly retweeted are extracted in Table 12. Among the discussion topics, the most common was scam, followed by mistrust, side effects and inefficiency.

### 4.5. Vaccine Hesitancy Analysis Using N-Grams

An n-gram analysis was conducted using the scikit-learn [39] Python library on the *cleaned* set. The purpose of this analysis was to obtain both additional insights and to uncover any additional relevant topics. As a preliminary step, the tweets were preprocessed by removing urls, converting multiple spaces to a single space, removing special characters and converting the text to a lowercase representation. The stop words included in the list provided by the NLTK [31] library were removed.

The extracted types of n-gram combinations include unigrams (1-g), bigrams (2-g) and trigrams (3-g) [40]. For each type, the 15 most relevant n-grams in order of appearance, have been selected by excluding the n-grams which do not bring any insights regarding the COVID-19 vaccine hesitancy reasons.

#### 4.5.1. Unigrams

The selected unigrams are presented in Table 13. It can be observed that most of the unigrams are in the areas of side effects, inefficiency, scam, mistrust and existence of alternatives. Popular terms, such as “effects”, “side”, “effective” and “risk” appear more than 3000 times in the considered dataset.

#### 4.5.2. Bigrams

In the case of bigrams, the selected terms are included in Table 14. Among the most common topics, one can identify “side effects” (2738 times), “long term” (1281 times) and “herd immunity” (917 times).

Considering the nine topics listed in Section 4.3.1, based on the bigrams analysis, it can be observed that the main discussion topics are side effects, existence of alternatives, mistrust, inefficiency and scam.

#### 4.5.3. Trigrams

The selected top-15 trigrams are presented in Table 15. The trigrams mainly belong to the following discussion topics (with respect to the discussion topics identified in 4.3.1): side effects, existence of alternatives, mistrust and scam.

Based on the n-gram analysis it can be observed that the discussions revolved around the following four main topics: side effects, existence of alternatives, mistrust and scam.

In all the three cases (unigrams, bigrams and trigrams) the main topic was represented by the presence of side effects, as a result of the COVID-19 vaccination process.

Another discussion topic that was identified was inefficiency, which has been observed in the case of unigrams and bigrams. For unigrams, “effective” and “efficacy” appear 5377 times, highlighting the importance of this topic in the context of vaccination hesitancy.

### 4.6. Vaccine Hesitancy Analysis Using Latent Dirichlet Allocation

Latent Dirichlet allocation (LDA) [41] is an unsupervised statistical approach for automatically discovering topics in a corpus. It relies on the bag-of-words paradigm and word-document counts to generate groups composed of several terms, which suggest a common shared topic. Over time, it has been applied for determining the main topics in different situations, such as e-petition content analysis [42], consumer complaints [43], determining information needs in health communities [44], analysis of railroad accident text [45], detecting micro-blog hot topics [46], etc. In COVID-19 context, Abd-Alrazaq et al. [47] identified 12 discussion topics, including the impact on the economy, the origin of the virus and approaches that can be applied for reducing the risk of contagion, by analyzing a tweet dataset collected from the beginning of February 2020 to mid-March 2020 for COVID-19. The authors have shown that LDA can be successfully used in such contexts.

In our case, in order to identify the main discussion themes related to vaccine hesitancy, the *cleaned* dataset, containing only the tweets classified as *against* vaccination, has been analyzed using LDA.

Given the fact that the collected tweets contain many tokens that do not provide any useful information and can interfere with the topic discovery, a preprocessing step was employed. The preprocessing approach was slightly different from that performed in the case of stance classification, given the different inner workings and purpose of the algorithms. The alterations that were performed on the text of the tweets are discussed in the following section.

As a first step, emoticons were removed with the help of the emoji (https://github.com/carpedm20/emoji, accessed on 20 September 2021) Python package, while urls and punctuation were removed using regular expressions. Then, the text was converted to lower case and divided into separate tokens by employing the NLTK library. The general stop words provided by NLTK were removed, as well as corpus specific keywords, including various terms by which users refer to COVID-19, as well as words related to politics. Last, the tokens were supplemented by the addition of the bigrams that appeared more than 10 times in the corpus.

The topic discovery was implemented with the help of the “gensim” (https://radimrehurek.com/gensim, accessed on 20 September 2021) library [48]. The analysis was performed considering both the original form of the tokens, as well as applying lemmatization, implemented through the spaCy (https://spacy.io, accessed on 20 September 2021) library, which led to better results.

**Figure 9:**
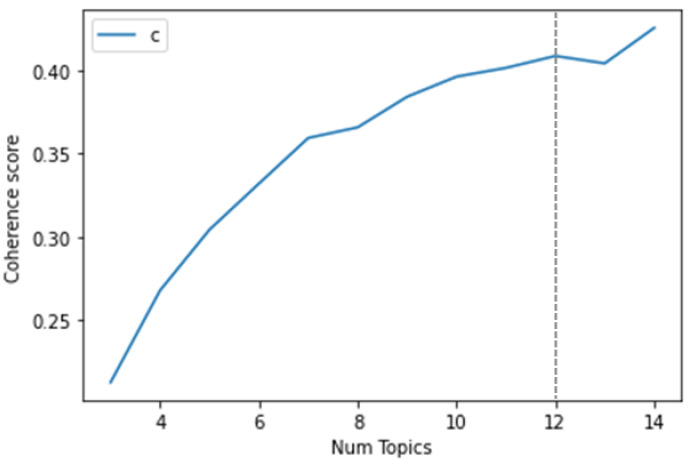
Relation between the number of topics and the coherence score.

In order to choose the number of topics that will be used in the LDA analysis, the coherence score was computed, while varying the number of topics between 3 and 14, as shown in Figure 9. As can be observed, an adequate coherence, while limiting the number of topics, is achieved when using 12 topics.

Afterwards, additional fine-tuning of the algorithm’s parameters was performed by varying the number of passes, which specifies the number of times the algorithm processes the documents in the corpus during the training phase [49]. In our case, the number of passes was varied in the interval 1 to 50, with 40 being chosen as an optimum value.

**Figure 10:**
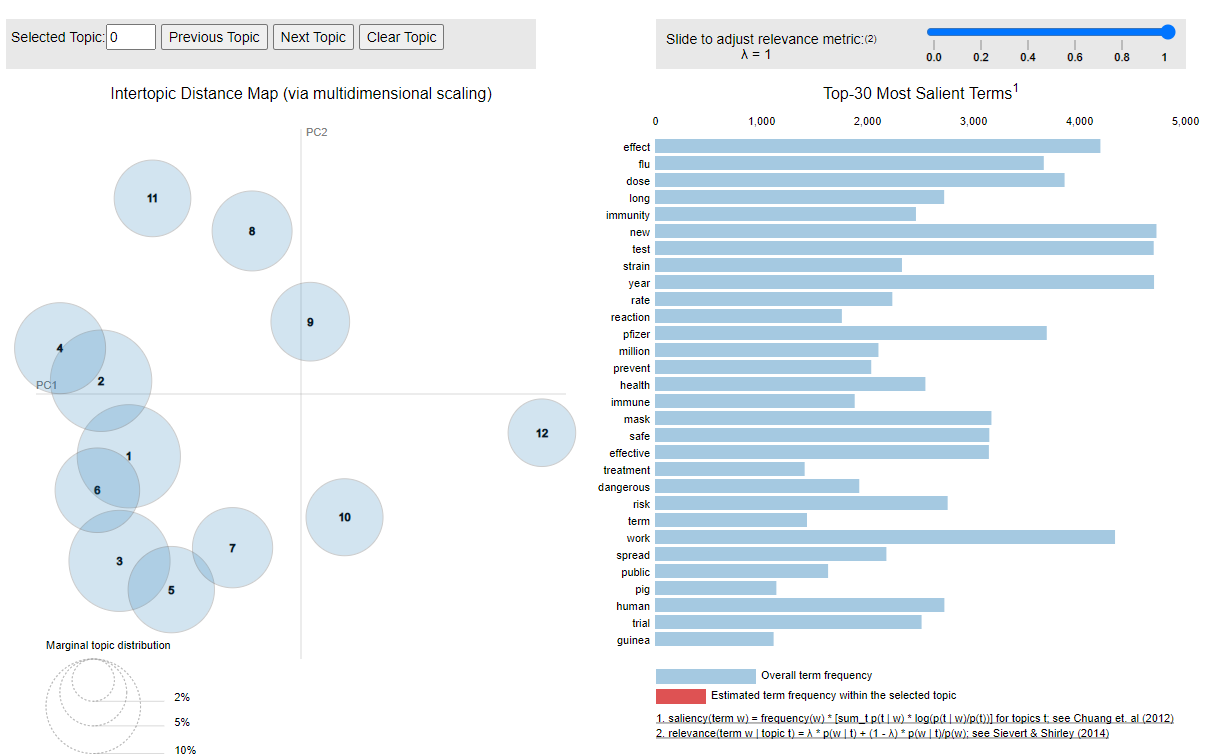
LDA topics and salient terms.

The visualization of the 12 identified topics is included in Figure 10, generated with the help of the pyLDAvis (https://pyldavis.readthedocs.io, accessed on 20 September 2021) library. The figure also includes the 30 most salient terms, as defined by Chuang et al. [50].

The results obtained using LDA are summarized in the first two columns of Table 16 and put into connection with the discussion topics identified through hashtag analysis (in Section 4.3.1).

Based on the discussion topics identified in Table 16 it can be observed that mistrust and side effects were the most encountered topics related to vaccine hesitancy. Among the reasons included in the mistrust topic one can mention: the longer period of time that would have been required for creating a safe vaccine, the inexistence of vaccines for other medical conditions, the fact that the health workers have refused to take the vaccine and that the vaccine is basically tested on the persons who accept vaccination. As for the side effects topic, the highlighted reasons are related to the appearance of long-term effects, allergic reactions, infertility, sterilization, autoimmune diseases and even death.

Additionally, discussion topics related to the existence of alternatives and scam were encountered as well in the analyzed set. For the existence of alternatives, it can be observed that the social media users mentioned two approaches, one related to the usage of ivermectin in preventing and treating the infection, while the second one emphasizing the development of the natural and heard immunity at a global level.

Lastly, hiding relevant information, inefficiency and freedom topics were also identified. The users’ discourse mainly focused on the appearance of new strains, hiding relevant scientific information and respecting one’s decisions related to her/his own body.

Overall, through the LDA analysis, 7 of the 9 discussion topics highlighted in Section 4.3.1 have been observed. The missing topics were related to unsafety and moral and religious issues.

## 5. Discussions and Limitations of the Study

Considering the analyses performed on the vaccine hesitancy datasets it can be observed that the variation in the number of tweets posted was in connection with the major events reported by the news in the corresponding days, which underlines the fact that people react through the use of the tweets to the news concerning COVID-19 vaccination. The so called “reaction” of the tweets to the news was in line with the previous research from the field, as observed in the studies conducted by D’Andrea et al. [28] regarding vaccination and by Tavoschi et al. [51] regarding the introduction of the mandatory immunization in Italy in 2016 for selected childhood diseases, which produced an increase of the social discussions on Twitter.

In terms of stance analysis, it was observed that in the analyzed period the opinions of Twitter users underwent some changes when compared to the one-month period [3] preceding the interval considered in this paper. Regarding the evolution of the *against* tweets in the *cleaned* datasets, a decrease from 17% to 6.78% was reported, while on the *entire* dataset, the decrease was from 10% to 4.4%. In absolute terms, the number of *against* tweets in the *cleaned* dataset decreased by 36,376 tweets (from 119,247 to 82,871), while in the case of the *entire* dataset the number of tweets decreased by 28,942 (from 250,294 to 221,352), showing a milder speech against COVID-19 vaccination.

Regarding the nine main discussion topics identified in the hashtags analysis, namely mistrust, freedom, side effects, hiding relevant information, unsafety, inefficiency, existence of alternatives, scam and moral and religious issues, it can be stated that they were in accordance with the findings of the other studies considering tweets analysis regarding COVID-19 vaccination hesitancy conducted on different periods of time during the pandemic.

For example, in a study conducted by Nuzhath et al. [52] in the one-month period between 19 July 2020–19 August 2020, previous to the vaccine announcement, the authors identified seven discussion topics related to: misinformation, safety and efficacy, conspiracy theories, mistrust of scientists and governments, lack of intent to get a vaccine, freedom of choice and religious beliefs.

Another study on Twitter, conducted between 9 December 2020–8 January 2021, on Turkish-related data identified 14 discussion topics [53]: poor scientific process, conspiracy theories, suspicion towards manufacturers, suspicion towards health authorities, undirected distrust, violation of autonomy, unsafety, non-necessary, ineffectiveness, people who are vaccinated or not, pandemic denial, financial concerns, membership of a specific ethnic group and religious beliefs.

Comparing the discussion topics in the pre-vaccination period considered in [52] with the discussion topics in the post-vaccination period included in our study and in [53], it can be observed that the main discussion lines stayed the same, which highlights even more the importance of the message transmitted by the authorities and the involved medical staff, as well as the need for a proper vaccine communication plan for better addressing people’s concerns. Moreover, the misinformation and conspiracy theories should be better debunked by the involved persons and social media platforms should be considered as a source for the spreading of this type of information.

The study has some limitations. Despite the popularity of Twitter, the users of this platform represent only a part of the English speakers all around the world and might not be representative for the English-speaking population as a whole. The identification of the COVID-19 vaccine tweets is strictly dependent of the keywords we have used in the study and the inclusion of different words might have conducted to a distinct dataset. The classification of the tweets in the three categories is also dependent on the accuracy levels of the model used, which, in some of the cases might not completely understand ironic phrases, easily detectable by a human. As Giachanou and Crestani [54] and Tavoschi et al. [51] pointed out, the automatic detection of irony and sarcasm in text can be a difficult task due to the fact that the presence of such a language can change the phrase to the opposite meaning. The period of time represents a limitation for the study as the results strictly refer to the selected period. By extending the period, the results of the analysis can be altered.

## 6. Conclusions

In this paper a Twitter analysis was performed by considering the tweets posted in the one-month period following the beginning of the COVID-19 vaccination process. Using a machine learning approach the tweets were divided into three main categories, according to their stance (*in favor*, *neutral* and *against*).

As we are mainly interested in the vaccine hesitancy reasons, the *against* tweets were analyzed in depth, by examining them in connection with the major events which took place during the considered period, through hashtag and top six retweets analysis, n-grams and LDA topic discovery.

Based on the COVID-19 hesitancy tweets analysis, nine main discussion topics were identified in the hashtags analysis: mistrust, freedom, side effects, hiding relevant information, unsafety, inefficiency, existence of alternatives, scam and moral and religious issues. A part of these topics (namely seven) were confirmed through LDA analysis, while only four discussion topics were identified when also considering n-gram analysis: side effects, existence of alternatives, mistrust and scam.

The results of the study can be applied in the context of addressing COVID-19 vaccine hesitancy concerns, as it identifies the main discussion topics and some of their underlying causes. This can be useful to the interested parties in the process of addressing the main fears and concerns of the persons who avoid or delay COVID-19 vaccination.

The work can be extended by considering a longer period of time and observing how the identified discussion topics evolve over time. Correlation analysis can be made in order to better shape the connection between the news posted in media and the tweets evolution.

The paper is accompanied by the n-grams and the annotated dataset which can be accessed at the following link: https://github.com/liviucotfas/covid-19-vaccination-hesitancy.

## Figures and Tables

**Table 1:** Keywords used for tweets searching.

Topic	Keywords
COVID-19	covid19, covid-19, coronavirus, coronaoutbreak, coronaviruspandemic, wuhanvirus, 2019nCoV
Vaccination	vaccine, vaccination, vaccinate, vaccinating, vaccinated

**Table 2:** The evolution of the number of tweets published in the analyzed period.

**Date**	**8 December**	**9 December**	**10 December**	**11 December**	**12 December**	**13 December**	**14 December**	**15 December**
***All***	308,125	184,086	131,142	116,808	144,513	138,021	277,660	201,788
***Cleaned***	71,010	53,983	45,382	40,110	39,492	33,323	65,271	53,108
**Date**	**16 December**	**17 December**	**18 December**	**19 December**	**20 December**	**21 December**	**22 December**	**23 December**
***All***	150,483	173,216	230,574	251,940	195,399	226,866	175,563	142,566
***Cleaned***	43,437	47,837	57,189	43,622	34,366	45,742	42,015	33,765
**Date**	**24 December**	**25 December**	**26 December**	**27 December**	**28 December**	**29 December**	**30 December**	**31 December**
***All***	84,690	44,926	63,180	99,702	106,641	151,111	261,538	183,485
***Cleaned***	23,252	13,478	15,993	21,430	26,899	36,187	51,687	35,975
**Date**	**1 January**	**2 January**	**3 January**	**4 January**	**5 January**	**6 January**	**7 January**	**TOTAL**
***All***	130,737	148,633	119,354	151,456	185,273	146,156	105,234	5,030,866
***Cleaned***	27,065	32,442	29,077	41,915	45,281	39,741	31,620	1,221,694

**Table 3:** Statistics for the manually annotated dataset.

Class	Number	Percentage
*against*	364	9.95%
*neutral*	2642	72.25%
*in favor*	651	17.80%
TOTAL	3657	100.00%

**Table 4:** Classification performance.

Code	Classifier	Class	Precision	Recall	F-Score	Accuracy
C1	**MNB** n-gram: (1, 2) features: 3000	*against*	67.26%	80.65%	73.28	**70.53%**
		*neutral*	75.70%	68.14%	71.64	
		*in favor*	70.18%	62.82%	66.18	
C2	**MNB** n-gram: (1, 3) features: all	*against*	66.01%	83.96%	73.88	69.77%
		*neutral*	82.20%	59.71%	69.11	
		*in favor*	67.05%	66.97%	66.93	
C3	**RF** n-gram: (1, 2) features: all	*against*	66.94%	77.53%	71.79	**68.53%**
		*neutral*	68.93%	72.50%	70.59	
		*in favor*	70.77%	55.56%	62.10	
C4	**RF** n-gram: (1, 3) features: all	*against*	66.93%	76.29%	71.23	67.86%
		*neutral*	67.25%	72.57%	69.77	
		*in favor*	70.60%	54.73%	61.52	
C5	**SVM** n-gram: (1, 2) features: all	*against*	73.31%	76.77%	74.90	**72.19%**
		*neutral*	74.20%	73.19%	73.63	
		*in favor*	69.34%	66.62%	67.86	
C6	**SVM** n-gram: (1, 3) features: all	*against*	70.35%	79.19%	74.39	71.73%
		*neutral*	76.03%	70.29%	72.99	
		*in favor*	69.77%	65.72%	67.53	
C7	**BERT** cased: no	*against*	78.96%	77.30%	77.97	**76.84%**
		*neutral*	77.82%	79.16%	78.35	
		*in favor*	74.29%	74.13%	74.07	
C8	**BERT** cased: yes	*against*	77.18%	76.25%	76.47	75.63%
		*neutral*	77.07%	77.06%	76.88	
		*in favor*	73.60%	73.92%	73.45	
C9	**RoBERTa**	*against*	76.82%	83.65%	79.87	**78.63%**
		*neutral*	81.82%	76.30%	78.84	
		*in favor*	78.23%	76.09%	76.99	

**Table 5:** Examples of *against* tweets.

Tweet Id	Text
1338493386949615616	If Donald and staff not taking covid-19 vaccine why would Anyone take it 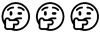
1339364438466433024	@Haminations COVID-19 is a hoax made by the government and the vaccine will be implanting micro chips in our bodies. There, I can never be wrong again.
1336679283230695424	@mariannaspring Either way, this vaccine is crap, covid is no worse than the flu.covid-19 create to impose the new world order!
1345658691874390017	@AdvoBarryRoux There still can’t produce a vaccine for HIV & Cancer, and all of a sudden there’s a vaccine 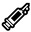 for COVID-19 in just one year it’s a trap
1338509542607425537	If I don’t take the flu shot every year then I don’t need the vaccine. Immune system strong over here https://t.co/7z3fXy6lbl
1345700961294102528	This is a TRAIL “vaccine” how can they distribute that much when it is NOT yet proven? 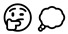 Normal citizens we are on our own. https://t.co/fC2b0dMXr5
1337373755702726657	@itvnews If this vaccine makes you immune to COVID19 then why would anyone having been vaccinated be at risk of catching COVID19??? Unless of course there’s something else in this vaccine that they want to inject every human on this Planet with??? https://t.co/VYzwf0yZCw
1336779512558854148	I have severe allergies for which I carry an Epipen and have had to use them a few times. The announcement was made to warn people like me to not take the Pfizer vaccine. I will wait for the next one thanks. https://t.co/iHBiD8dUmS

**Table 6:** Top-3 specific hashtags for the *against* tweets.

*Cleaned* Dataset		*All* Dataset	
#novaccineforme	266	#billgatesbioterrorist	565
#bigpharma	223	#covidvaccinesideeffects	560
#scamdemic	155	#malefertility	477

**Table 7:** Discussion topics and issues for #novaccineforme.

Discussion Topics	Issues
Mistrust	It has not yet been possible to produce a vaccine for human immunodeficiency virus (HIV) and diabetes for years;
	The incapacity of producing a vaccine for an unknown virus;
	Not tested enough;
	More research needed;
	Healthcare workers don’t vaccinate themselves;
Freedom	The freedom of choice (“my body, my choice”);
	Rights violation;
	Freedom is not for sale;
Side effects	Death;
	Health damages;
	Adverse outcomes;
	Allergies;
	Sterilization;
	Potential alteration of deoxyribonucleic acid (DNA);
	Side-effects similar to COVID-19 which does not justify the vaccination: high fever, malaise, difficulty in breathing, severe muscle aches, loss of smell, chills;
Hiding relevant information	Experts have been banned;
	The exact ingredients are proprietary and are not publicly disclosed;
	Low trust in the data provided by the authorities;
Unsafety	No guarantee that the vaccine is safe;
	The state does not offer injury compensation to the ones who have suffered adverse health effects caused by vaccines;
Inefficiency	It only protects 9 from 1000 persons;
Existence of alternatives	Natural medicine;
	Ivermectin;
	Trust in immune system which offers a 99.9% protection versus vaccine 95%;
Scam	Important public figures have not been vaccinated yet;
	The videos with vaccinating public figures are fake;
	Depopulation;
	People would become reliant on vaccines and anti-viral medication;
	Vaccine is the whole purpose of this inexistent pandemic;
Moral and religious issues	Vaccines are made from aborted fetuses.

**Table 8:** Discussion topics and issues for #bigpharma.

Discussion Topics	Issues
Mistrust	Humans are not guinea pigs;
	The need for many years to create a vaccine;
	Not properly tested;
Side effects	Risk of autoimmune disorders;
	Short- and long-term adverse effects;
	5 volunteers died after taking the vaccine;
	A Portuguese nurse died after receiving the vaccine;
	Vaccines are harmful;
Hiding relevant information	Aluminum as an adjuvant component;
	The need for an independent immunologist or virologist for explaining how the vaccine works;
	No data available on how vaccine works;
	No data available related to the vaccine ingredients;
	The burying of the adverse effects data;
Unsafety	Cannot be sued for wrongful injury;
Existence of alternatives	Ivermectin;
	Vitamin C;
	Hydroxychloroquine;
	Leronlimab;
	Zinc;
	Virus with a 99.5% recovery rate;
Scam	Complete control of population;
	Money/profit for the pharmaceutical companies;
	Contains a protein that encodes bioluminescence which can genetically modify organisms in order to monitor them (in a quantity of 66.6 mL);
	Corrupt politicians;
	COVID-19 vaccine is just a placebo, the politicians on TV have been vaccinated with sugar water.

**Table 9:** Discussion topics and issues for #scamdemic.

Discussion Topics	Issues
Mistrust	Refuted by peer review reports;
	The existence of doctors who explain the negative aspects;
	Vaccine is associated with an experimental poison;
	mRNA is not even considered experimental, but theoretical;
	No old people have participated in experimental studies;
Side effects	Causes sterilization in girls and young woman;
	Causes infertility by autoimmune response targeting placenta during pregnancy;
	Bell’s Palsy disease can be an effect of the vaccination;
	Increased risk of contracting HIV/ acquired immunodeficiency syndrome (AIDS);
	Changes the DNA;
	Severe allergic reactions;
Hiding relevant information	Unknown vaccine ingredients;
Unsafety	No legal prosecution for the vaccine producers;
Existence of alternatives	Heard immunity as the solution for the pandemic;
	The virus is not more deadly than the flu, so no treatment is needed;
	Hydroxychloroquine;
	Vitamin C;
	Vitamin D;
	Z-Pak;
	Zinc;
	World leaders have been treated using other treatments (no vaccine for them);
Scam	Population control and depopulation is the purpose of the vaccination process;
	The TV vaccinated persons have been vaccinated with empty syringes;
	The vaccination has the purpose to make a natural selection;
	The entire pandemic is a hoax;
	It is an experiment for reprogramming human DNA;
	Pharmaceutical companies earn billions;
	COVID-19 death totals are exaggerated;
Moral and religious issues	The vaccine contains pork gelatin;
	The vaccine contains DNA from aborted fetuses.

**Table 10:** Discussion topics and issues for #billgatesbioterrorist.

Discussion Topics	Issues
Side effects	Adverse effects in general;
	Testing positive for HIV after vaccination;
	Male fertility;
Scam	Inserting microchips into human bodies;
	Earning money from the vaccination process;
	Population control/depopulation/population reduction.

**Table 11:** Discussion topics and issues for #covidvaccinesideeffects.

Discussion Topics	Issues
Mistrust	Humans are not guinea pigs;
	Not properly tested;
Side effects	700,000 deaths or disabilities as result of vaccination;
	Brain bleeds or strokes;
	Severe allergic reactions;
	Anaphylactic reactions;
	Bell’s Palsy;
	Examples of persons passing out after COVID-19 vaccination;
	Examples of persons dying after COVID-19 vaccination;
	Toxic effects due to the use of mRNA;
	Male/female fertility;
Hiding relevant information	Not knowing the risks of vaccines;
Unsafety	The pharmaceutical companies are protected from responding for the side effects;
	No liability for any side effects;
Existence of alternatives	Trust in the immune system.

**Table 12:** Top six retweets.

Tweet Id	Retweets	Text
1339418638189813761	5198	Twitter allowed the Russian collusion hoax, plus the Brett Kavanaugh gang-rapist hoax to circulate...They censored the TRUE Hunter Biden story and will now censor anything that goes against the multi-billion dollar, evil, Covid-19 ring. I will not be touching the vaccine. https://t.co/3sFZcCBNQM
1343710964391284736	4424	The World Health Organization has just stated there is no evidence yet that vaccines will prevent #COVID19 infections and therefore stop the transmission of the virus. In that case, why are we doing this?
1339818851123400707	2024	Nurse faints immediately after taking experimental Covid-19 vaccine. Rushed experimental biological agents like this should not be mandated upon anyone. Meaning airlines, employers, schools, nor the government can ever tell anyone else they must take an experimental vaccine! https://t.co/UIelzjE6Sh
1337069198661611522	1968	The first 2 people in the UK to get the shot went into anaphylactic shock basically. Now they’re advising anyone with allergies not to take the shot, and when giving the shot to have resuscitation equipment available! When will we learn (They) are trying to kill us! No Vaccines! https://t.co/gyUsjAEEVo
1336216657837035520	1610	As a pharmacist/medic who has developed medicines for Big Pharma for 25+ years, I can categorically say that this vaccine is NOT necessary + not even effective. Covid-19 fraud is now being revealed and people WILL go to prison for crimes against humanity: https://t.co/uxzkVmDOOb
1345300003661615104	1218	I am not going to get vaccinated for now. How can I trust BJP’s vaccine, when our government will be formed everyone will get free vaccine. We cannot take BJP’s vaccine: Samajwadi Party chief Akhilesh Yadav#COVID19 https://t.co/qnmGENzUBH

**Table 13:** Top 15 selected unigrams.

Unigrams	Number of Appearances
effects	3969
side	3518
effective	3177
risk	3071
efficacy	2200
dangerous	1858
strain	1852
death	1782
hoax	1625
experimental	1401
rushed	1380
die	1289
hiv	1259
allergic	1025
ivermectin	747

**Table 14:** Top 15 selected bigrams.

Bigrams	Number of Appearances
side effects	2738
long term	1281
herd immunity	917
immune system	856
stop risk	823
risk proven	823
guinea pigs	727
new strain	695
big pharma	666
side effect	526
common cold	499
allergic reactions	426
experimental vaccine	373
guinea pig	316
rushed vaccine	312

**Table 15:** Top 15 selected trigrams.

Trigrams	Number of Appearances
stop risk proven	823
long term effects	389
99 survival rate	372
side effects covid	281
vaccine based greed	228
rush vaccine based	227
expect rush vaccine	227
greed instead research	227
hydroxychloroquine saves lives	226
vaccine side effect	218
repurposed generics suppressed	178
dose ivermectin malaria	177
generics suppressed fraudulently	177
understand side effects	174
treatment ivm works	171

**Table 16:** LDA topics, keywords and discussion topics.

Topic Extracted Using LDA	Keywords Included	Discussion Topic
Topic 1	year, mrna, develop, cure, research, test, hiv, cancer	Mistrust
Topic 2	hoax, lie, fake, control, kill, bill, lockdown, wear_mask, evil, money, believe	Scam
Topic 3	test, risk, trial, die, old, child, death, woman, result, safe, false, cause, clinical_trial	Side effects
Topic 4	big, pharma, hell, big_pharma, conspiracy, trust, needle, inject, president, government, fake, million	Scam
Topic 5	dose, pfizer, science, fda, moderna, biontech, experiment, article, work, trust, protection, research	Hiding relevant information
Topic 6	refuse, worker, health, care, government, healthcare, hospital, nurse, trust, nhs, doctor, medical	Mistrust
Topic 7	flu, new, strain, rate, mutate, chance, effective, mutation, survival, new_strain, survival_rate, efficacy	Inefficiency
Topic 8	immunity, public, health, heard, heard_immunity, experimental, natural, harm, immune	Existence of alternatives
Topic 9	effect, long, spread, long_term, transmission, term, infertility, sterilization, autoimune	Side effects
Topic 10	dangerous, mandatory, force, right, refuse, mandate, choice, school, mask, travel, body, cdc, isolate	Freedom
Topic 11	treatment, safe, effective, dose, ivermectin, solution, safe_effective, fraudently_discredit, safety, prevent	Existence of alternatives
Topic 12	reaction, pig, guinea, allergic, guinea_pig, severe, allergic_reaction, allergy, kill, adverse, adverse_action, danger, human_guinea	Side effects, Mistrust

## Data Availability

The n-grams and the annotated dataset are available at: https://github.com/liviucotfas/covid-19-vaccination-hesitancy.
